# Esophageal Perforation Resulting From Nonaccidental Trauma in a Neonate: A Case Report

**DOI:** 10.7759/cureus.74819

**Published:** 2024-11-30

**Authors:** Lanah Almatroud, Rashed Hasan

**Affiliations:** 1 College of Human Medicine, Michigan State University, East Lansing, USA; 2 Pediatrics, Hurley Medical Center, Flint, USA

**Keywords:** child abuse, infant respiratory distress, nonaccidental injuries, pediatrics & neonatology, traumatic esophageal perforation

## Abstract

Esophageal perforation (EP) resulting from nonaccidental trauma in a neonate is extremely rare. We report a previously healthy 12-day-old neonate presenting with stridor, respiratory distress, and bloody vomitus. Clinical, radiographic, and endoscopic evaluations confirmed the diagnosis of EP. The patient received respiratory support, remained on nothing by mouth (NPO) status, and was administered parenteral nutrition for seven days until healing was confirmed. Upon resuming oral feeding, the neonate tolerated it well and was discharged home. A follow-up endoscopy, six weeks later, revealed normal findings. Early recognition and treatment of EP are crucial to prevent complications. To our knowledge, this is the youngest reported case of EP due to nonaccidental trauma.

## Introduction

Esophageal perforation (EP) is rare in the United States, with an estimated incidence of three cases per 100,000 individuals [1]. EP in infants and children occurs even less frequently than in adults. When it occurs, it is often associated with instrumentation of the aerodigestive tract, including attempts at tracheal intubation, suctioning of the upper aerodigestive tract, and endoscopic or surgical procedures involving the upper gastrointestinal tract [2]. Other causes of EP in children include trauma, ingestion of caustic substances or foreign bodies, and complications related to endoscopic procedures [3,4]. In infants and children, the most common clinical signs of EP include respiratory distress with stridor and vomiting, which may be accompanied by hematemesis. Other reported symptoms at initial presentation include fever, poor feeding, and difficulty breathing [5]. However, these symptoms are nonspecific and can delay the diagnosis, increasing the risk of severe complications. Imaging, particularly computed tomography (CT) scans and contrast esophagography, plays a vital role in identifying EP [6], and can also reveal associated complications, such as pneumomediastinum and mediastinitis [1]. Despite significant advancements in diagnostic modalities and therapeutic interventions, EP remains a life-threatening condition, with mortality rates reported as high as 50% in some studies [1]. EP resulting from nonaccidental trauma in neonates is exceedingly rare, with only one prior case reported in a neonate [7].

We present the case of a previously healthy 12-day-old neonate who was brought to the Emergency Department (ED) with respiratory distress and hematemesis. Clinical history, physical examination, imaging, and endoscopic findings were consistent with EP secondary to nonaccidental trauma. Our case emphasizes the importance of early recognition and management of EP to prevent complications, such as mediastinitis and mediastinal abscess formation. Early diagnosis, through high clinical suspicion and prompt imaging or endoscopic evaluation, is critical to improving outcomes in neonates with suspected EP [1,8,9]. To our knowledge, this is the youngest reported case of EP resulting from nonaccidental trauma. The abstract of this article was previously presented at the ACG's 2024 Annual Scientific Meeting & Postgraduate Course in Philadelphia, Pennsylvania, USA, on October 27, 2024. 

## Case presentation

A 12-day-old neonate was brought to the ED with stridor, respiratory distress, and bloody vomitus. The mother reported being woken by the infant’s biological father shouting profanities as he forcibly inserted a feeding bottle into the baby’s mouth. She observed blood coming from the infant’s mouth and noted difficulty breathing. The neonate was born to a healthy gravida 1, para 1 mother at 36 weeks of gestation via spontaneous vaginal delivery. Apgar scores were 6 at one minute and 7 at five minutes. He experienced respiratory distress at birth and was managed with continuous positive airway pressure (CPAP) for 36 hours before successfully weaning to ambient air. An umbilical pH of 7.1 was noted. A nasogastric tube (NGT) was placed without difficulty, and a plain radiograph confirmed its proper positioning. The NGT was used for enteral feeding for three days, after which it was removed. The neonate tolerated oral feeding well and was discharged on room air five days after birth.

**Figure 1:**
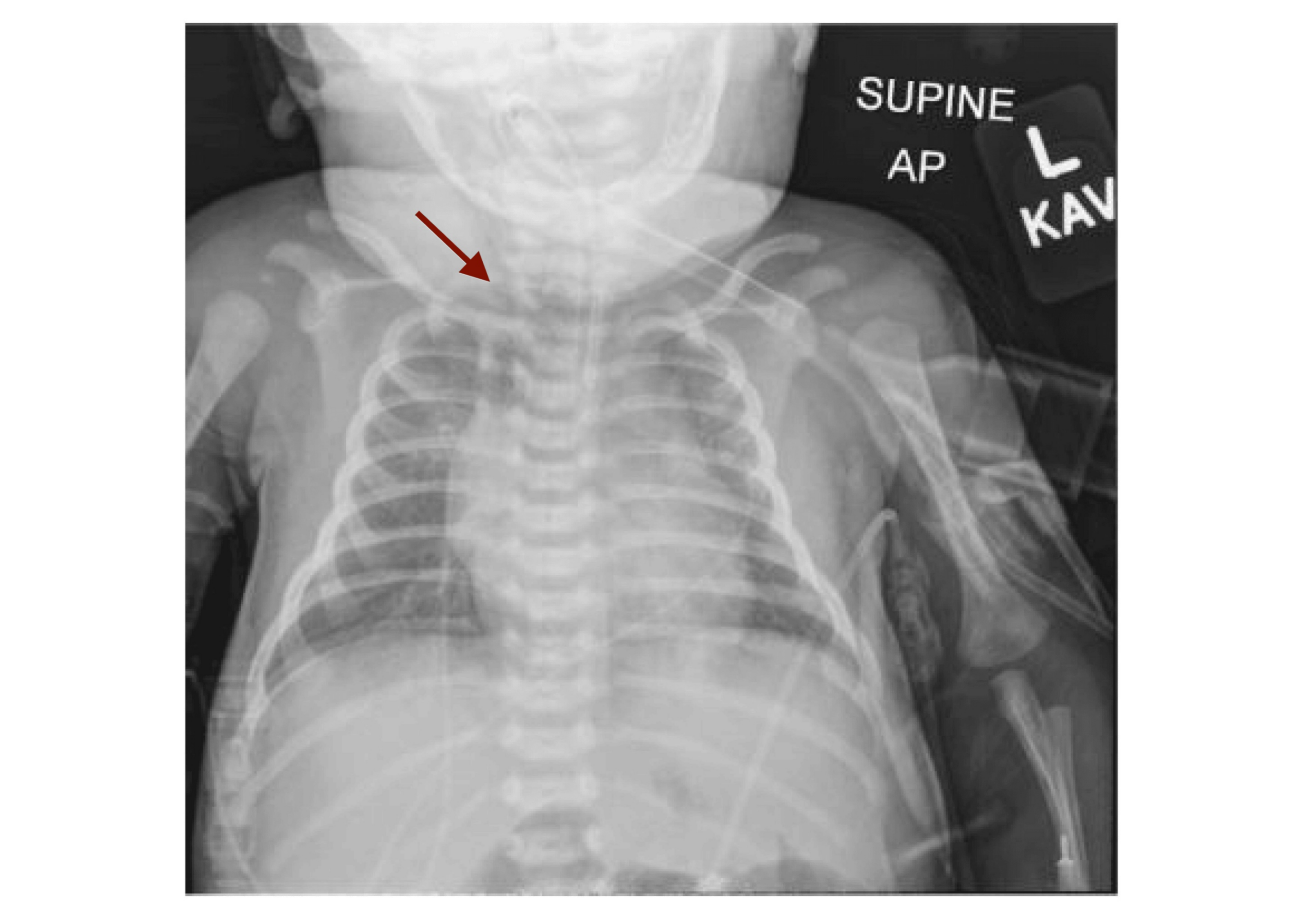
Anteroposterior chest radiograph showing pneumomediastinum (arrow) immediately following intubation.

**Figure 2:**
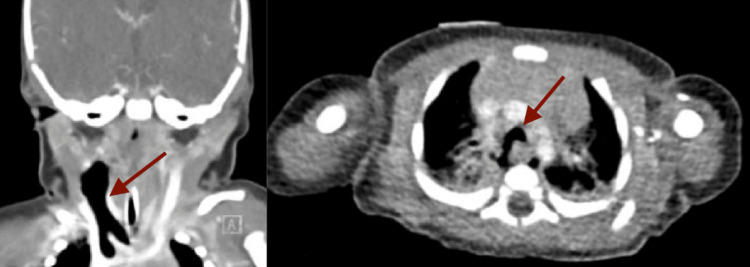
Computed tomography (CT) scan showing extensive air dissecting (arrows) through the soft tissues of the mediastinum and neck.

**Figure 3:**
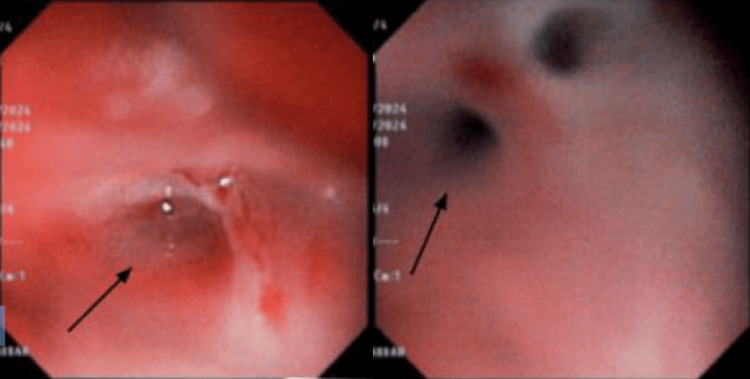
Endoscopy showing a false tract (arrow) originating from the upper one-third of the esophagus, with fresh blood at the edges. The normal esophagus is seen at the 1 o’clock position, with no evidence of injury.

At the time of presentation to the ED, the neonate exhibited inspiratory stridor. His vital signs included a respiratory rate of 55 breaths per minute, a heart rate of 155 beats per minute, a temperature of 37.1°C, oxygen saturation of 95% on ambient air, and blood pressure of 95/50 mmHg. Fresh bloody vomitus was observed despite the lack of deep suctioning or instrumentation by Emergency Medical Services (EMS). There was no evidence of external trauma, and the oral cavity and oropharynx appeared normal. The neonate was intubated on the first attempt using a size 0 Miller blade and a size 3.0 cuffed endotracheal tube without complications. During intubation, the pharynx and larynx, including the vocal cords, appeared normal, with no signs of trauma. A chest radiograph obtained immediately after intubation showed evidence of pneumomediastinum (Figure 1). A CT scan revealed extensive air dissecting through the soft tissues of the mediastinum and neck (Figure 2). Endoscopy identified a false tract originating from the esophagus, in addition to the normal esophageal opening (Figure 3).

Skeletal survey and retinal examinations were performed to assess for other signs of nonaccidental trauma and were both normal. Coagulation studies were notable for a prothrombin time (PT) of 10.3 seconds, partial thromboplastin time (PTT) of 26.5 seconds, and an international normalized ratio (INR) of 1, all of which were within normal limits. The neonate was kept nothing by mouth (NPO) and started on parenteral nutrition. Mechanical ventilation was continued. Child Protective Services (CPS) mandated that the father have only supervised visits with the neonate. After seven days, esophagography showed no extravasation of contrast, indicating healing of the esophagus. The baby’s trachea was extubated, and oral feeding was reintroduced, which he tolerated well. He was discharged home two days later. Follow-up esophagography and endoscopy performed six weeks later were both normal.

At the age of four months, the baby was seen in the ED and was subsequently hospitalized for multiple bruises on the lower neck, chest, and abdomen. The father admitted to inflicting these injuries. This subsequent presentation raised further concerns about nonaccidental trauma, highlighting the need for ongoing vigilance in such cases.

## Discussion

EP resulting from nonaccidental trauma is exceedingly rare, and its occurrence in neonates due to child maltreatment is even less common [7]. EP typically presents with respiratory distress and subcutaneous emphysema, and its symptoms may resemble those of other serious conditions, such as myocardial infarction [10]. However, clinical manifestations may be subtle or absent early in the disease course, necessitating a high index of suspicion for prompt diagnosis [11]. Prompt diagnosis and tailored interventions are critical in managing EP, as morbidity and mortality are influenced by the corrosive nature of gastrointestinal contents and the extent of bacterial contamination in the paraesophageal spaces [12]. Several anatomical and histological factors contribute to the clinical significance of EP. The absence of a serosal layer, and the esophagus’s encasement in only loose areolar tissue, facilitate the leakage of bacteria and digestive enzymes into the mediastinum, increasing the risk of severe mediastinitis, empyema, sepsis, and ultimately multi-organ system failure [2]. Clinicians must be aware of the esophagus's unique anatomical features and remain vigilant to identify EP as early as possible in its course.

Only one other documented case of neonatal EP caused by nonaccidental trauma has been reported in the literature. Pramuk et al. reported a case of a 15-day-old neonate who presented with copious oral secretions due to a nonaccidental traumatic tear of the posterior aspect of the upper esophagus. The injury was promptly identified, and the neonate was managed with NPO status, leading to complete healing of the esophagus by three months of age without residual damage [8]. In our case, the presence of fresh blood from the neonate's mouth - without prior instrumentation of the upper aerodigestive tract - and normal oropharyngeal and laryngeal findings raised concerns for an esophageal injury. The identification of pneumomediastinum further heightened our clinical suspicion. The upper gastrointestinal endoscopy led to an early diagnosis of this serious issue. Early identification of the esophageal injury and maintaining the patient on NPO status likely minimized the risk of complications. This rare case emphasizes the critical need for vigilance when evaluating neonatal respiratory distress. Identifying unusual cases, such as nonaccidental trauma, and implementing timely management can result in favorable outcomes, even in severe injuries.

## Conclusions

EP in neonates, especially from nonaccidental trauma, is an exceptionally rare and life-threatening condition. Prompt recognition and intervention are crucial to prevent severe complications, such as mediastinitis and sepsis. This case underscores the importance of considering esophageal injury in neonatal respiratory distress with atypical findings and reinforces the need for healthcare providers to remain vigilant for signs of nonaccidental trauma in vulnerable patient populations. Early diagnosis, supportive care, and a multidisciplinary approach can significantly improve outcomes in these critical cases.
